# Navigating Time-Inconsistent Behavior: The Influence of Financial Knowledge, Behavior, and Attitude on Hyperbolic Discounting

**DOI:** 10.3390/bs14110994

**Published:** 2024-10-24

**Authors:** Aliyu Ali Bawalle, Sumeet Lal, Trinh Xuan Thi Nguyen, Mostafa Saidur Rahim Khan, Yoshihiko Kadoya

**Affiliations:** School of Economics, Hiroshima University, 1-2-1 Kagamiyama, Higashihiroshima 739-8525, Japan; d231201@hiroshima-u.ac.jp (A.A.B.); lsumeet@hiroshima-u.ac.jp (S.L.); nguyen93@hiroshima-u.ac.jp (T.X.T.N.); ykadoya@hiroshima-u.ac.jp (Y.K.)

**Keywords:** hyperbolic discounting, time-inconsistent behavior, financial literacy, intertemporal preferences, financial wellbeing

## Abstract

Hyperbolic discounting is a psychological phenomenon in which individuals prioritize smaller immediate rewards over larger future rewards. Time-inconsistent behavior is deemed irrational as it negatively impacts savings and investment, investment in financial knowledge, and long-term financial and personal well-being. This study hypothesizes that improving financial knowledge, promoting positive financial behavior, and fostering a future-oriented financial attitude can mitigate hyperbolic discounting bias and that these three components of financial literacy enable investors to make long-term economic decisions maximizing utility. We analyzed the responses of 114,170 active investors in Japan to examine the interactions between financial knowledge, behavior, and attitude. Our findings reveal a strong negative relationship between these dimensions and hyperbolic discounting, underscoring their crucial role in shaping individuals’ intertemporal preferences. For researchers, our results highlight the need to integrate multidimensional aspects of financial literacy into investigations of intertemporal discounting behaviors. Policymakers should implement holistic financial education programs that improve knowledge, transform behavior, and shape attitudes. Financial institutions and advisors should prioritize programs that mitigate hyperbolic discounting tendencies among clients. This study represents a significant advancement in the research on financial literacy, offering a comprehensive framework for future studies and practical applications aimed at improving financial decision-making outcomes.

## 1. Introduction

Hyperbolic discounting is a psychological phenomenon that prioritizes smaller immediate rewards over larger future rewards when the waiting period is closer to the present than the future, leading to time-inconsistent behavior [1,2,3,4,5,6,7]. Time-inconsistent behavior is considered irrational [2]. Hyperbolic discounting not only decreases saving and investment behavior but also negatively affects investment in financial knowledge [8], which, in turn, adversely affects people’s financial and personal well-being later in life. Understanding the factors that reduce hyperbolic discounting can help individuals, financial advisors, and policymakers to formulate strategies that will reduce its consequences. While Katauke et al. [9] examined the association between hyperbolic discounting and financial literacy using measures of financial knowledge, few studies have investigated the role of financial behavior and financial attitudes. To address this gap in the literature, our study expands the scope by incorporating these dimensions into our model. We hypothesize that enhancing financial knowledge, promoting positive financial behavior, and fostering a future-oriented financial attitude can reduce hyperbolic discounting bias. These components empower individuals to make more informed and beneficial financial decisions, contributing to more rational financial behaviors [10,11].

Previous studies have provided various theoretical explanations for why hyperbolic discounting leads to suboptimal economic and financial decisions in the long run. First, according to time-inconsistency theory, hyperbolic discounting leads to time-inconsistent preferences, where an individual’s preferences change over time in ways that their earlier selves would disapprove. This inconsistency can result in a failure to follow long-term plans, such as saving for retirement or adhering to a health regimen [12,13]. When future benefits are excessively devalued, individuals may opt for choices that provide immediate satisfaction but lower overall utility over time. Second, commitment problem theory suggests individuals often struggle with self-control and commitment due to time-inconsistent preferences. They may plan to take actions that benefit their future selves but fail to execute these plans when the time comes. For example, some might intend to save a portion of their income each month but spend it on immediate pleasures [14]. This lack of commitment to long-term goals reduces overall utility, since short-term desires consistently override long-term benefits. Third, intertemporal tradeoff theory posits that hyperbolic discounting distorts intertemporal tradeoffs, causing individuals to undervalue future rewards and overvalue immediate ones. This distortion can result in long-term suboptimal choices. For example, a person might choose to indulge in immediate consumption rather than saving, which results in lower wealth accumulation and less financial security in the future [13,15]. Theories on present bias and myopic preferences also illustrate how individuals make investment and savings decisions that produce short-term gains [16,17] that lead to over-consumption, procrastination, and insufficient investment in long-term projects [13,18].

This study is based on the assumption that the three components of financial literacy—knowledge, behavior, and attitude—enable investors to make long-term economic and financial decisions that maximize their utility [10,11,19,20,21,22,23]. Individuals with financial knowledge are better equipped to manage financial resources, evaluate financial products and services, and respond appropriately to events that may adversely affect their financial well-being [11,24,25]. In addition to the influence of financial knowledge on investment and financial decisions, financial behavior and attitude also have an impact on long-term value-maximizing economic and financial decisions. For instance, studies have linked financial behaviors to financial satisfaction [26,27], financial well-being [22], student income [28], and self-esteem [29]; moreover, financial attitudes have been associated with rational choices, such as retirement planning [21], savings [30], and financial management [31,32]. The behavior of young investors when making investment decisions is influenced by their financial knowledge, attitude, and behaviors [33,34,35]. However, few studies have examined the influence of financial behavior and attitude on hyperbolic discounting, despite the fact that these factors can greatly influence investors’ time-inconsistent behavior. Additionally, the influence of financial behavior and attitude on hyperbolic discounting could be distinct despite their relevance to each other [10,11,36,37,38,39]. Therefore, unraveling the discrete influence of these components provides a more nuanced understanding of the interplay between rational behavior, proxied by the three components of financial literacy and hyperbolic discounting.

Explaining hyperbolic discounting from the viewpoint of financial knowledge, behavior, and attitude paves the way for developing strategies to mitigate its effects. The mitigation process works through three channels. First, financial knowledge, positive financial behavior, and a future-oriented financial attitude act as commitment devices for future-oriented behaviors such as automatic savings plans or investment accounts with withdrawal restrictions [40]. Second, financial literacy helps investors to understand the benefits of long-term savings and investment [40]. Finally, long-term savings, investment, retirement planning, and the timely payment of debt are embedded in financial literacy concepts and subtly guide individuals toward making better long-term decisions without restricting their freedom of choice. Examples include default options for retirement savings and timely reminders of debt repayments [41].

This study contributes to the literature in several ways. First, this is the first study to provide empirical evidence on the influence of financial behavior and attitude on hyperbolic discounting while reaffirming the existing evidence on the negative association between financial knowledge and hyperbolic discounting. Second, we analyze the responses of 114,170 active investors from one of Japan’s leading online securities companies. Using a large dataset of Japanese investors allows us to comprehensively examine the interactions between financial knowledge, behavior, and attitude. Third, we provide policymakers, financial institutions, and researchers with recommendations on how to reduce the negative effects of hyperbolic discounting on economic decisions.

The remainder of this paper is organized as follows. In Section 2, we present the data and a summary of the statistics, hypotheses, and methods. Section 3 presents the empirical results; the results are discussed in Section 4. Section 5 concludes the paper and provides policy implications.

## 2. Materials and Methods

The data were collected from a 2023 Money and Life survey jointly conducted by Rakuten Securities, one of the leading online security companies in Japan, and Hiroshima University, Japan. The survey targeted Rakuten Securities clients who were over 18 years old and had logged into the company’s online website at least once since 2022. The online survey was administered by sending an email containing the survey questions to all account holders from November to December 2023. The questionnaire was designed to obtain information on the demographic, socioeconomic, and psychological characteristics of the Japanese population, as well as their financial skills and investment performance. After excluding respondents missing sociodemographic information, the final sample consisted of 114,170 respondents, representing 60.21% of the total observations. We used this extensive dataset to examine the association between financial knowledge, behavior, attitudes, and hyperbolic discounting among Japanese individuals.

Our primary dependent variable is hyperbolic discounting, which reflects the degree to which future gains (delayed rewards) are valued less than immediate rewards, demonstrating the influence of present bias and impulsivity on household decision making. Groundbreaking research in behavioral economics has shown that individuals tend to apply higher discount rates to shorter time horizons while using lower rates for longer horizons [16,42]. To measure hyperbolic discounting, we adopted the methodology of Kang et al. [3] and Ikeda et al. [43]. Specifically, we used two intertemporal choice questions, detailed in Appendix A. These questions assessed the preference of the respondents to receive varying amounts of money over different time delays. In Question 1, participants chose between options over a 2–9 day period, while in Question 2, they chose over a 90–97 day period. For each question, respondents selected between 8 option As and 8 corresponding option Bs, with the hypothetical monetary reward increasing incrementally. The measurement of hyperbolic discounting follows a two-step process [3,43,44]. First, discount rates were elicited by identifying the “switching point” where respondents shifted their preference from option A to option B. Based on these switching points, we calculated discount rates 1 and 2 (DR1 and DR2) and used a binary classification to identify hyperbolic discounters. A value of 1 was assigned to hyperbolic discounters (where DR1 > DR2) and 0 to non-hyperbolic discounters. This approach is consistent with the studies by Fukuda et al. [44], Katauke et al. [9], and Zhang [7]. The concept of hyperbolic discounting has practical relevance in various real-world settings, particularly in health behaviors and financial decision making. For instance, studies by Kang and Ikeda [3,45] have shown that present-biased preferences can result in short-term decisions that undermine long-term goals, such as excessive smoking or poor health choices. Their findings suggest that individuals who heavily discount future rewards may face difficulties in sustaining healthy behaviors, contributing to issues like higher smoking rates or obesity. Similarly, Ikeda et al. [43] linked hyperbolic discounting to behaviors such as debt accumulation and poor financial planning, underscoring the challenge individuals face in balancing immediate gratification with long-term financial stability.

Since this study aims to comprehensively explore how the three components of financial literacy affect hyperbolic discounting, the primary independent variables are financial knowledge, financial behavior, and financial attitude. To measure the variables, we followed the methodology of Kadoya and Khan [10] using self-reported responses to several questions and statements. Three independent variables were constructed as continuous variables, with values ranging from 0 to 1. Detailed descriptions of the coding are as follows. First, financial knowledge evaluates respondents’ understanding of basic financial terms and concepts, such as inflation, interest rates, and risk diversification, which allows them to compare financial products and services and make prudent and informed financial decisions. We measured financial knowledge using three questions related to basic calculations that account for interest rates, inflation rates, and the understanding of investment risks (see Appendix B). Providing the correct answers to the financial knowledge questions requires a basic understanding of inflation, risk diversification, and the ability to perform simple and compound interest arithmetic. We assigned one point for correct responses and zero for incorrect responses to the three financial knowledge questions. A financial knowledge index was then constructed by computing the average scores of the financial knowledge questions.

Second, financial behavior illustrates an individual’s actions and inactions in financial transactions, reflecting how they translate their financial knowledge into real-life financial decisions. For instance, positive financial behaviors, such as saving, paying credit on time, budgeting, careful buying, and bargaining, improve individual financial freedom and foster household financial well-being. We measured financial behavior using four types of questions related to buying, paying bills, and financial planning. For each question, a point was assigned based on the extent to which respondents agreed to the statements displayed on a Likert scale, where 1 = Applies and 5 = Not at all. We assigned one point to respondents who selected 1 = Applies or 2 = Somewhat true and zero otherwise (see Appendix B). A financial behavior score was obtained by averaging the individual scores from each of the four questions. Third, financial attitude is a measure of people’s outlook on financial issues [10]. Financial attitude is considered a catalyst for financial decisions. For instance, financial knowledge and the ability to act rationally in financial transactions are only feasible in the presence of an appropriate financial attitude. We constructed financial attitude scores by assessing the degree to which two statements on money spending and living in the moment (see Appendix B) applied to the respondents. These qualitative statements were evaluated on a 5-point Likert scale, where 1 = Applies and 5 = Not at all. Based on this scale, we assigned one point to those who chose 4 = Not very applicable and 5 = Not at all and zero otherwise. As higher scores indicate a positive attitude, we averaged the scores assigned to the two statements to obtain the final financial attitude score of the respondents. Furthermore, we measured Cronbach’s alpha to assess the reliability of the financial behavior and financial attitude scales. The results, though not shown here to save space, indicate alpha values of 0.69 for financial behavior and 0.63 for financial attitude, suggesting that the reliability of both scales is marginally acceptable.

Furthermore, we controlled for the possible effects of socioeconomic and behavioral factors in our regression analysis to corroborate the impact of financial knowledge, financial behavior, and financial attitude on hyperbolic discounting. Detailed descriptions of the variables are presented in Table 1.

### 2.1. Descriptive Statistics

This study’s statistics are summarized in Table 2. The respondents’ mean scores and standard deviations (SDs) for financial knowledge, financial behavior, and financial attitude were 0.794 (0.297), 0.768 (0.286), and 0.647 (0.374), respectively. About 11% of the respondents were hyperbolic discounters, and 53% were risk averse. It is important to note that the percentage of respondents exhibiting hyperbolic discounting is significantly lower than that found in the general population (61.1%) [43]. This difference is likely due to the fact that the respondents in this study are investors, who tend to be more forward-looking and focused on long-term outcomes than the average individual. Among the study population, 60% were male, 67% were married, and 69% had a university degree. Respondents had an average age of 44, which is slightly higher than the average age of 42 years reported in a similar study conducted with Japanese adults [46]. Approximately 95% of the respondents were employed, 45% had children, and 6% were divorced. On average, respondents earned JPY 7.57 million per year and had accumulated JPY 18.71 million in total financial assets.

Table 3 presents the distribution of hyperbolic discounting by age, gender, marital status, and employment. We found a statistically significant difference in the results. For example, approximately 12.26% of male respondents were hyperbolic discounters, as opposed to 10.07% of female respondents. The proportion of married respondents was 11.70%, while the proportion of nonmarried respondents was 10.78%. In addition, 11.27% of employed respondents reported being hyperbolic discounters, as opposed to 13.93% of unemployed respondents. Furthermore, the findings revealed a generational gap in hyperbolic discounting tendency. Those 65 years and older had a higher proportion of hyperbolic discounters (15.12%), while 11.31% of respondents aged 40 to 65 and 10.69% of respondents under the age of 40 were hyperbolic discounters. Although the higher proportion of hyperbolic discounters among respondents aged 65 and older (15.12%) compared to younger age groups is counterintuitive, the phenomenon could be explained by several factors related to aging. Cognitive control tends to decline with age, making it more difficult for older adults to delay gratification and resist immediate rewards, which is characteristic of hyperbolic discounting. Additionally, older individuals often have shorter perceived time horizons, leading them to prioritize short-term benefits over long-term rewards. Increased uncertainty about health and financial stability in later life can also make immediate consumption more attractive, further contributing to this trend [43].

In addition, we used a t-test to examine the mean variation in hyperbolic discounting with respect to financial knowledge, behavior, and attitude. The results showed mean differences in financial knowledge, behavior, and attitudes; specifically, between respondents with high financial knowledge (M = 0.78, SD = 0.31) and low financial knowledge (M = 0.80, SD = 0.79), the result was significant (t = 3.71 *p* = 0.0001). In addition, there was a significant difference between respondents with higher financial behavior (M = 0.60, SD = 0.39) and those with lower financial behavior (M = 0.65, SD = 0.37) (t = 13.90, *p* = 0.0000). Furthermore, the mean value was lower for respondents who reported higher financial attitudes (M = 0.74, SD = 0.29) than those with poorer financial attitudes (M = 0.77, SD = 0.28) (t = 11.74, *p* = 0.0000), indicating that the mean difference was significant.

### 2.2. Methodology

We considered financial knowledge, behavior, and attitude as instruments for making rational decisions and investigated their influence on hyperbolic discounting. Hyperbolic discounting is a behavioral tendency that drives instant gratification. People with irrational dispositions, for example, prefer smaller rewards that are immediate over larger rewards that are delayed. According to Samuelson [44], individuals discount their future utility in a simple regular pattern, which means time preference is not present. Researchers have used the concept to explain intertemporal choices. Theoretically, according to Samuelson [47], an outcome with utility A at t = 0 would be valued at $A.\delta^{t}$ at $t_{t+1}$. As a result, the present time value (V) of future utility can be determined using the following equation:

(1) $$V\left(A,t\right)=A.\delta^{t}$$

where δ  is a time-invariant discount rate, 0≤δ≤1.

Several studies have shown that exponential discounting cannot explain some individuals’ behavior. Due to such anomalies, economic theories on behavioral decisions about intertemporal choice have switched from exponential discounting to hyperbolic discounting [5]. The hyperbolic discounting function is given by

(2) $$w\left(D\right)=\frac{1}{\left(1+kD\right)}$$

where k is the discountrate, k>0, $w\left(D\right)$ is the discount factor that multiplies the benefits, *D* is the delay period, and *k* is the parameter governing the degree of discounting.

$V\left(D\right)$ is the subjective value of the reward available at time *D*, and $D\left(0\right)$ is the subjective value of the available reward at time *D* = 0.

After constructing the hyperbolic discounting function, we classified the respondents into hyperbolic and non-hyperbolic discounters, and since the outcome variable is binary, we used a probit regression to estimate the following functional equation:

(3) $$Y_{i}=f\left({Fin\_Know}_{i},{or\;{Fin\_Beh}_{i}\;or\;{Fin\_Att}_{i}X}_{i},\varepsilon_{i}\right)$$

where $Y_{i}$ indicates whether respondent *i* is a hyperbolic discounter or not, Fin_Know , Fin_Beh, and Fin_Att  represent financial knowledge, financial attitude, and financial behavior, respectively, $X_{i}$ is a vector of ${individual}_{i}$ characteristics, and $\varepsilon_{i}$ is the error term. The full specifications of Equation (3) are presented below:


(3a) $$\begin{array}{c} Hyperbolic\;discounting\\ \;\;\;\;\;\;=\alpha +\beta_{1}{Financial\;knowledge}_{i}+\beta_{2}{Gender}_{i}+\beta_{3}{Marriage}_{i}\\ \;\;\;\;\;\;+\beta_{4}{Age}_{i}+\beta_{5}{Employment}_{i}+\beta_{6}{Divorce}_{i}+\beta_{7}{Childreen}_{i}\\ \;\;\;\;\;\;+\beta_{8}{Education}_{i}+\beta_{9}{Household\;Income}_{i}\\ \;\;\;\;\;\;+\beta_{10}{Household\;Asset}_{i}+\beta_{11}{Risk\;Aversion}_{i}+\varepsilon \end{array}$$

(3b) $$\begin{array}{c} Hyperbolic\;discounting\\ \;\;\;\;\;\;=\alpha +\beta_{1}{Financial\;behavior}_{i}+\beta_{2}{Gender}_{i}+\beta_{3}{Marriage}_{i}\\ \;\;\;\;\;\;+\beta_{4}{Age}_{i}+\beta_{5}{Employment}_{i}+\beta_{6}{Divorce}_{i}+\beta_{7}{Childreen}_{i}\\ \;\;\;\;\;\;+\beta_{8}{Education}_{i}+\beta_{9}{Household\;Income}_{i}\\ \;\;\;\;\;\;+\beta_{10}{Household\;Asset}_{i}+\beta_{11}{Risk\;Aversion}_{i}+\varepsilon \end{array}$$

(3c) $$\begin{array}{c} Hyperbolic\;discounting\\ \;\;\;\;\;\;=\alpha +\beta_{1}{Financial\;attitude}_{i}+\beta_{2}{Gender}_{i}+\beta_{3}{Marriage}_{i}\\ \;\;\;\;\;\;+\beta_{4}{Age}_{i}+\beta_{5}{Employment}_{i}+\beta_{6}{Divorce}_{i}+\beta_{7}{Childreen}_{i}\\ \;\;\;\;\;\;+\beta_{8}{Education}_{i}+\beta_{9}{Household\;Income}_{i}\\ \;\;\;\;\;\;+\beta_{10}{Household\;Asset}_{i}+\beta_{11}{Risk\;Aversion}_{i}+\varepsilon \end{array}$$

### 2.3. Multicollinearity Assessment

To ensure that the three key predictors in this study, financial knowledge (FK), financial attitude (FA), and financial behavior (FB), captured the distinct dimensions of financial decision making and were not redundant, we conducted a multicollinearity assessment. This step was critical, as the predictors may be interrelated, potentially leading to biased estimates in the regression models. Multicollinearity was tested using the variance inflation factor (VIF). The VIF values quantify how much the variance of a regression coefficient is inflated due to multicollinearity among the independent variables. According to conventional guidelines, a VIF value greater than 10 indicates problematic multicollinearity, and values between 5 and 10 may signal moderate collinearity issues. The results of the VIF test for all independent variables, that is, FK, FA, and FB, control variables, and the dependent variable indicated no signs of multicollinearity, with all VIF values well below the commonly accepted threshold of 2 (Appendix C: Table A2). This finding supports the conclusion that each predictor, that is, FK, FA, and FB, represents a distinct aspect of financial decision making. Consequently, no variable serves as a proxy for another, allowing us to proceed with confidence in the probit regression analysis.

For further confirmation of this, an intercorrelation matrix was calculated for FK, FA, FB, and hyperbolic discounting (HD) (Appendix C: Table A1). While these variables are intercorrelated to some extent, none of the pairwise correlations approached the threshold for multicollinearity (typically correlations above 0.7 or 0.8). The highest correlation was 0.360, well below the level of concern. This further validated the absence of multicollinearity, as these moderate correlations confirmed that the predictors were not excessively interrelated. As a result, we concluded that the regression estimates derived from these predictors were not biased due to multicollinearity.

## 3. Empirical Results

We used probit regression models to explain the link between financial knowledge, behavior, attitudes, and hyperbolic discounting. In the analysis, we constructed three models: model 1 used financial knowledge, model 2 used financial behavior, and model 3 focused on financial attitude as the main variable of interest. Gender, marital status, age, employment, divorce status, children, university education, household income, household assets, and risk aversion were incorporated into all the models.

Table 4 reports the probit regression coefficients that explain hyperbolic discounting. Specifically, models 1, 2, and 3 contain the regression coefficients for financial knowledge, financial behavior, and financial attitude, respectively. The results of models 1, 2, and 3 show a significant negative association between financial knowledge, financial behavior, financial attitude, and hyperbolic discounting. In model 1, we found that being male, married, and older was positively related to hyperbolic discounting. Meanwhile, the findings show a negative relationship with employment. Furthermore, we found no relationship between divorce, having children, university education, household income, household assets, risk aversion, and hyperbolic discounting. The signs and magnitudes of the covariates in models 2 and 3 are consistent with those of model 1.

Additionally, we conducted a subsample analysis using gender and age. The results of the subsample analysis are presented in Table 5 and Table 6. The results in Table 5 show that female respondents are more likely to be hyperbolic discounters when financial knowledge or financial behavior is the main explanatory variable, and male respondents are more likely to be hyperbolic discounters when financial attitude is the main explanatory variable, and the results are statistically significant. Regarding age, the results of Table 6 indicate the absence of a significant relationship for respondents aged 60 and older. However, there is evidence of a negative and significant association with hyperbolic discounting for respondents aged 40 to 60 and under 40 years of age. Regardless of whether the main explanatory variable is financial knowledge, behavior, or attitude, the signs and significance are the same; however, the magnitude increases from financial knowledge to financial behavior and financial attitude.

## 4. Discussion

Despite the importance of understanding the interaction between hyperbolic discounting and financial knowledge, behavior, and attitude, this study is the first to explore this association. To bridge this gap in the literature, we investigated the dynamic interaction between hyperbolic discounting and the three components of financial literacy. Such an examination is crucial because it sheds light on how a greater understanding of financial knowledge, behavior, and attitude can help us devise strategies to mitigate the effects of cognitive biases such as hyperbolic discounting. The insights gained could have significant policy implications for improving financial management and economic well-being.

This study’s main findings provide evidence of a negative association between financial knowledge, behavior, and attitude and hyperbolic discounting. In particular, the results suggest that financial knowledge can reduce hyperbolic discounting, consistent with the findings of Katauke et al. [9]. Our findings also support the studies linking lower levels of financial literacy with indecisiveness and inconsistency in time-preference choices [48,49]. Our findings suggest individuals can behave rationally and make optimal decisions by understanding basic financial concepts and the implications of inflation and compound interest. Moreover, our findings indicate that financial behavior is significantly and negatively associated with hyperbolic discounting. Although there is little empirical evidence of an association between hyperbolic discounting and financial behavior, some studies have explored the role of financial behavior with other forms of cognitive bias. Although various cognitive biases may differ in their manifestation, they may represent deviations from rational judgment [50]. We used the findings of previous studies to infer and validate our findings. For example, studies have shown that overconfidence, a cognitive bias, often results in suboptimal economic behaviors such as excessive market entry and overborrowing [51,52]. These biases can affect an individual’s ability to rationally assess the long-term consequences of financial decisions. In contrast, our research suggests that through a robust understanding of financial concepts, bias can be mitigated by fostering better financial habits, such as greater self-control and patience [53]. This enhanced financial behavior would enable people to make informed and deliberate financial decisions, thereby reducing the impulse to discount future rewards.

Similarly, we provide evidence that financial attitude mitigates hyperbolic discounting tendencies. This finding is consistent with previous studies that have shown the impact of cognitive biases on financial attitudes. For instance, research has shown that personality traits can predict cognitive biases moderated by risk attitude [54] and that a lower cognitive ability is linked to financial optimism [55]. Our findings suggest a similar link between financial attitude and hyperbolic discounting. We argue that just as cognitive biases can be influenced by financial attitudes, hyperbolic discounting can also occur. This supports the notion that good financial attitudes, underpinned by financial education, can help individuals make better financial decisions and minimize the impact of cognitive biases such as hyperbolic discounting [20]. Overall, our findings reveal a significant negative association between financial knowledge, behavior, and attitude and hyperbolic discounting, underscoring the pivotal role of these factors in promoting prudent financial decisions and mitigating the propensity for short-term gratification. This highlights the importance of improving financial literacy and fostering positive financial behaviors and attitudes to curb hyperbolic discounting tendencies, ultimately leading to improved financial well-being.

One important consideration in interpreting our findings is the generalizability of the specific indicators used to measure financial attitude (FA) and financial behavior (FB). In this study, FA and FB were measured following the OECD methodology [11,56,57], using indicators related to specific financial actions, such as living in the present and careful spending. These measures capture the key aspects of financial decision making related to household debt, savings, and future financial security. However, while these indicators reflect critical financial behaviors, they may not fully generalize to all financial contexts. Specifically, the chosen measures focus on common household financial challenges, such as debt management and savings, but may not encompass more complex behaviors like investment strategies, retirement planning, or long-term wealth accumulation. Therefore, caution is needed when extrapolating these results to broader financial patterns. Although the findings align with the literature linking attitudes and behaviors to financial outcomes [46], further research should explore whether these relationships hold in different financial behaviors and more diverse financial contexts.

Regarding the demographic and socioeconomic variables, our results suggest that hyperbolic discounting is significantly and positively related to age. These findings are consistent with the findings of Katauke et al. [9], Zhang [7], and Richards et al. [58]. Furthermore, the results show gender differences in hyperbolic discounting. For instance, women appear to be less hyperbolic than men, and our results align with the findings of Dittrich and Leipold [59], Richards et al. [58], and Meier et al. [60]. Additionally, we found that employment is negatively linked to hyperbolic discounting, consistent with the findings of Bassem and Mohamed [61] and Ikeda et al. [43]. One possible explanation for this result is the effect of employment on income, which could potentially minimize the intertemporal disposition suggested by Aimon et al. [62]. According to Robb and Woodyard [63], income has a significant impact on financial behavior, while a study by Green et al. [64] revealed that lower-income adults had a greater tendency to intertemporal discounting. Additionally, employment fosters a future-oriented mindset, as individuals plan for career growth and retirement. The structure and routine of a job also encourage self-discipline, which helps to delay gratification. Lastly, access to financial-planning tools, such as retirement plans, reinforces long-term thinking, further reducing impulsive decision making. Moreover, we found that marriage is positively associated with hyperbolic discounting, which is consistent with the findings of Bernedo et al. [65]. Marital relationships can accelerate or reduce impulsive behavior in several ways. For example, the different financial management styles of spouses can increase impulsive decisions. In addition, changes in either partner’s financial or mental well-being can trigger impulsive behaviors that cause future consumption to be sacrificed for immediate satisfaction. However, education was not found to have a significant association with hyperbolic discounting. Education may not be significantly related to hyperbolic discounting after controlling for financial knowledge, behavior, and attitude because these variables directly influence decision making more than education alone. While education can provide a general foundation, it does not always translate into financial literacy or behavioral change. Financial knowledge, in contrast, directly equips individuals with the tools and understanding necessary to make informed, future-oriented financial decisions, potentially reducing tendencies toward impulsivity. Similarly, financial behavior and attitude reflect how individuals actually handle their finances and their mindset toward long-term goals, which are more closely tied to hyperbolic discounting than formal education levels.

There are several limitations of this study that should be considered carefully while interpreting the results. First, the sequence in which the participants answered the questions could affect the responses to later questions. For example, financial behavior and attitude questions were presented before the hyperbolic discounting tasks, which could have influenced participants’ responses to the latter by unconsciously signaling their preferences. However, given the structure of the discounting tasks, such as varying time frames, multiple alternatives, and identifying switching points, we believe the likelihood of substantial bias is low. Second, while our sample included a large number of active investors, they were all drawn from a single company, which may limit the generalizability of our findings to broader investor populations. Finally, this study relied on self-reported measures for financial behavior and financial attitude, which can introduce social desirability or optimism bias. While the alignment between self-reported tendencies and discounting choices lends some validity, this could reflect self-perception rather than actual behavior. Future studies should incorporate objective behavioral data to address this potential bias. Additionally, longitudinal studies are needed to rigorously examine how hyperbolic discounting behaviors change under various economic conditions, providing a deeper understanding of the phenomenon in dynamic financial contexts.

## 5. Conclusions

This study extends the scope of the findings of Katauke et al. [9] by incorporating financial behavior and attitude. This innovative approach examines the influence of the three components of financial literacy on hyperbolic discounting behavior. In doing so, we provide a nuanced understanding of their separate impacts on hyperbolic discounting behavior, thus contributing to significant advancements in the field. To the best of our knowledge, this is the first study to examine the influence of financial knowledge, behavior, and attitude on hyperbolic discounting tendencies. We found a strong negative relationship between these dimensions and hyperbolic discounting, underscoring their crucial role in shaping individuals’ intertemporal preferences in financial decision making. Among the control variables, demographic and socioeconomic characteristics such as age, gender, employment, and marriage status were found to be significantly associated with hyperbolic discounting behavior.

These insights have substantial implications for academia, financial institutions, and policymakers. Our study emphasizes the need to integrate the multidimensional aspects of financial literacy into future investigations of intertemporal discounting behaviors. By comprehensively examining financial knowledge, behavior, and attitude, researchers can achieve a deeper understanding of their collective impact on decision-making processes across diverse contexts. Our findings underscore the potential benefits of implementing holistic financial education programs for policymakers. Such programs, which are designed to simultaneously enhance knowledge, transform behavior, and shape attitudes, could foster more informed and resilient financial decision making among individuals and communities. Policymakers should also introduce awareness programs to inform people about the long-term adverse effects of short-term gratification. For financial institutions and advisors, our study suggests prioritizing programs that can mitigate hyperbolic discounting tendencies among clients. Overall, this study represents a significant advancement in financial literacy research and offers a comprehensive framework for future studies and practical applications to improve financial decision making.

## Figures and Tables

**Table 1:** Variable definitions.

Variables	Definition
Hyperbolic discounting	The binary variable equals 1 if DR1 > DR2 and 0 otherwise.
Financial knowledge	Average score of the three financial knowledge questions
Financial behavior	Average score of the four financial behavior questions
Financial attitude	Average score of the two financial attitude questions
Gender	Binary variable, 1 = Male and 0 = Female
Age	Age of the respondent
Married	Binary variable equals 1 if the respondent is married and 0 otherwise
Divorced	Binary variable equals 1 if respondent is divorced and 0 otherwise
Children	Binary variable equals 1 if the respondent has at least one child and 0 otherwise
University education	Binary variable equals 1 if respondent has at least a university degree and 0 otherwise
Employment	Binary variable equals 1 if the respondent is employed and 0 otherwise
Household income	Natural log of household annual gross income in 2023
Household assets	Natural log of household net financial assets
Risk aversion	A continuous measure of the risk aversion levels of respondents extracted from the question: Usually when you go out, how high must the probability of rain be before you take an umbrella?

**Table 2:** Descriptive statistics of the study variables.

Variable	Mean	Std. Dev.	Min	Max
Hyperbolic discounting	0.114	0.318	0	1
Financial knowledge	0.794	0.297	0	1
Financial behavior	0.768	0.286	0	1
Financial attitude	0.647	0.374	0	1
Gender	0.608	0.488	0	1
Age	43.858	11.754	18	90
Married	0.679	0.467	0	1
Divorced	0.063	0.243	0	1
Children	0.453	0.498	0	1
University education	0.685	0.464	0	1
Employment	0.95	0.219	0	1
Household income	7,568,170.3	4,126,853.9	1,000,000	20,000,000
Household assets	18,689,389	23,202,909	2,500,000	100,000,000
Risk aversion	0.536	0.228	0	1
Observations: 114,170				

**Table 3:** Distribution of hyperbolic discounting by sociodemographic factors.

Hyperbolic Discounting	Male		Employment		Married		Age			
	Female	Male	No	Yes	No	Yes	<40	40–64	65+	Total
0	40,282	60,870	49,54	96,198	32,717	68,435	43,371	48,711	9070	101,152
	89.93%	87.74%	86.07%	88.73%	89.22%	88.30%	89.31%	88.69%	84.88%	88.60%
1	4512	8506	802	12,216	3951	9067	5189	6213	1616	13,018
	10.07%	12.26%	13.93%	11.27%	10.78%	11.70%	10.69%	11.31%	15.12%	11.40%
Total	44,794	69,376	5756	108,414	36,668	77,502	48,560	54,924	10,686	114,170
	100%	100%	100%	100%	100%	100%	100%	100%	100%	100%
	t = −11.3634 ***		t = 6.2008 ***		t = −4.5869 ***		F = 85.89 ***			

*** *p* < 0.01.

**Table 4:** Probit regression results.

Dependent Variable: Hyperbolic Discounting			
	Model 1	Model 2	Model 3
Financial knowledge	−0.121 ***		
	(0.017)		
Financial behavior		−0.212 ***	
		(0.017)	
Financial attitude			−0.193 ***
			(0.013)
Gender	0.115 ***	0.104 ***	0.095 ***
	(0.011)	(0.01)	(0.01)
Married	0.031 **	0.037 ***	0.041 ***
	(0.014)	(0.014)	(0.014)
Age	0.004 ***	0.003 ***	0.004 ***
	(0.001)	(0.001)	(0.001)
Employment	−0.058 **	−0.06 **	−0.054**
	(0.023)	(0.023)	(0.024)
Divorced	0.016	0.021	0.025
	(0.023)	(0.023)	(0.023)
Children	−0.001	0.000	0.002
	(0.012)	(0.012)	(0.012)
University education	0.003	0.001	−0.002
	(0.011)	(0.011)	(0.011)
Household income	0.000	0.000	0.000
	(0.000)	(0.000)	(0.000)
Household assets	0.000	0.000	0.000
	(0.000)	(0.000)	(0.000)
Risk aversion	0.026	0.033	0.033
	(0.021)	(0.021)	(0.021)
_Cons	−1.319 ***	−1.234 ***	−1.296 ***
	(0.037)	(0.038)	(0.037)
Observations	114,170	114,170	114,170
Pseudo R^2^	0.004	0.005	0.006

Robust standard errors are in parentheses. *** *p* < 0.01, ** *p* < 0.05.

**Table 5:** Gender subsample probit regression results.

	Female	Male	Female	Male	Female	Male
Financial Knowledge	−0.061 **	−0.178 ***				
	(0.025)	(0.024)				
Financial Behavior			−0.193 ***	−0.224 ***		
			(0.028)	(0.021)		
Financial Attitude					−0.208 ***	−0.185 ***
					(0.022)	(0.016)
Gender						
Married	0.006	0.044 **	0.012	0.051 ***	0.02	0.052 ***
	(0.023)	(0.018)	(0.023)	(0.018)	(0.023)	(0.018)
Age	0.003 ***	0.004 ***	0.003 ***	0.004 ***	0.003 ***	0.004 ***
	(0.001)	(0.001)	(0.001)	(0.001)	(0.001)	(0.001)
Employment	−0.093 **	−0.046 *	−0.092 **	−0.048 *	−0.089 *	−0.038
	(0.046)	(0.028)	(0.046)	(0.028)	(0.046)	(0.028)
Divorced	0.017	0.012	0.022	0.019	0.029	0.021
	(0.033)	(0.032)	(0.033)	(0.032)	(0.033)	(0.032)
Children	−0.007	0.002	−0.007	0.003	−0.002	0.004
	(0.019)	(0.015)	(0.019)	(0.015)	(0.019)	(0.015)
University Education	−0.011	0.01	−0.012	0.007	−0.013	0.002
	(0.018)	(0.015)	(0.018)	(0.014)	(0.017)	(0.014)
Household Income	0.000	0.000	0.000	0.000	0.000	0.000
	(0.000)	(0.000)	(0.000)	(0.000)	(0.000)	(0.000)
Household Assets	0.000	0.000 *	0.000	0.000	0.000	0.000
	(0.000)	(0.000)	(0.000)	(0.000)	(0.000)	(0.000)
Risk Aversion	−0.007	0.039	−0.001	0.047 *	0.001	0.047 *
	(0.038)	(0.026)	(0.038)	(0.026)	(0.038)	(0.026)
_cons	−1.278 ***	−1.186 ***	−1.169 ***	−1.156 ***	−1.202 ***	−1.244 ***
	(0.065)	(0.048)	(0.067)	(0.048)	(0.065)	(0.045)
Observations	44,794	69,376	44,794	69,376	44,794	69,376
Pseudo R^2^	0.001	0.003	0.003	0.004	0.004	0.004

Robust standard errors are in parentheses. *** *p* < 0.01, ** *p* < 0.05, * *p* < 0.1.

**Table 6:** Age group subsample probit regression results.

Variable	<40 Years	40–60 Years	65+ Years	<40 Years	40–60 Years	65+ Years	<40 Years	40–60 Years	65+ Years
Financial Knowledge	−0.151 ***	−0.088 ***	−0.043						
	(0.025)	(0.026)	(0.062)						
Financial Behavior				−0.259 ***	−0.207 ***	−0.047			
				(0.025)	(0.025)	(0.058)			
Financial Attitude							−0.235 ***	−0.178 ***	−0.081 *
							(0.02)	(0.019)	(0.043)
Gender	0.132 ***	0.11 ***	0.044	0.113 ***	0.105 ***	0.043	0.098 ***	0.099 ***	0.042
	(0.016)	(0.016)	(0.039)	(0.016)	(0.015)	(0.039)	(0.016)	(0.015)	(0.039)
Married	0.027	0.052 **	0.025	0.039 *	0.059 ***	0.026	0.045 **	0.059 ***	0.027
	(0.021)	(0.022)	(0.051)	(0.021)	(0.022)	(0.051)	(0.021)	(0.022)	(0.051)
Age	−0.004 **	0.006 ***	0.015 ***	−0.006 ***	0.006 ***	0.015 ***	−0.005 ***	0.006 ***	0.015 ***
	(0.002)	(0.001)	(0.004)	(0.002)	(0.001)	(0.004)	(0.002)	(0.001)	(0.004)
Employment	−0.075	−0.015	0.024	−0.059	−0.014	0.024	−0.062	−0.007	0.026
	(0.059)	(0.044)	(0.037)	(0.059)	(0.044)	(0.037)	(0.059)	(0.044)	(0.037)
Divorced	0.141 ***	0.017	−0.045	0.149 ***	0.022	−0.043	0.155 ***	0.024	−0.043
	(0.044)	(0.031)	(0.075)	(0.044)	(0.031)	(0.075)	(0.044)	(0.031)	(0.075)
Children	−0.004	0.01	−0.04	−0.005	0.013	−0.039	−0.001	0.014	−0.038
	(0.02)	(0.016)	(0.034)	(0.02)	(0.016)	(0.034)	(0.02)	(0.016)	(0.034)
University Education	−0.023	0.005	0.01	−0.03	0.004	0.01	−0.031 *	0.000	0.009
	(0.018)	(0.016)	(0.035)	(0.018)	(0.016)	(0.035)	(0.018)	(0.016)	(0.034)
Household Income	0.000	0.000	0.000	0.000	0.000	0.000	0.000	0.000	0.000
	(0.000)	(0.000)	(0.000)	(0.000)	(0.000)	(0.000)	(0.000)	(0.000)	(0.000)
Household Assets	0.000	0.000	0.000	0.000	0.000	0.000	0.000	0.000	0.000
	(0.000)	(0.000)	(0.000)	(0.000)	(0.000)	(0.000)	(0.000)	(0.000)	(0.000)
Risk Aversion	0.046	0.000	−0.022	0.05	0.008	−0.02	0.052	0.006	−0.015
	(0.034)	(0.03)	(0.071)	(0.034)	(0.03)	(0.071)	(0.034)	(0.03)	(0.071)
_cons	−1.03 ***	−1.541 ***	−1.972 ***	−0.902 ***	−1.464 ***	−1.976 ***	−0.983 ***	−1.522 ***	−1.972 ***
	(0.081)	(0.084)	(0.256)	(0.083)	(0.084)	(0.254)	(0.081)	(0.083)	(0.25)
Observations	48,560	54,924	10,686	48,560	54,924	10,686	48,560	54,924	10,686
Pseudo R^2^	0.003	0.003	0.003	0.005	0.004	0.003	0.006	0.004	0.003

Robust standard errors are in parentheses. *** *p* < 0.01, ** *p* < 0.05, * *p* < 0.1.

## Data Availability

The data that support the findings of this study were collected by Rakuten Securities in collaboration with Hiroshima University. These data are not publicly available due to restrictions under the licensing agreement for the current study. However, they can be made available from the authors upon reasonable request and with permission from Rakuten Securities and Hiroshima University.

## Appendix A. Intertemporal Questions

You were given a certain amount of money. You can get it after 2 or 9 days, but the amount will be different. If you had options A or B for the date and amount that you would receive, which would you choose? Choose whichever combination you like from 1 to 8 (only one of each).		
Question 1	Option A	Option B
Combination 1:	You will receive JPY 10,000 in 2 days.	After 9 days, you will receive JPY 9981.
Combination 2:	You will receive JPY 10,000 in 2 days.	After 9 days, you will receive JPY 10,000.
Combination 3:	You will receive JPY 10,000 in 2 days.	After 9 days, you will receive JPY 10,019.
Combination 4:	You will receive JPY 10,000 in 2 days.	After 9 days, you will receive JPY 10,038.
Combination 5:	You will receive JPY 10,000 in 2 days.	After 9 days, you will receive JPY 10,096.
Combination 6:	You will receive JPY 10,000 in 2 days.	After 9 days, you will receive JPY 10,191.
Combination 7:	You will receive JPY 10,000 in 2 days.	After 9 days, you will receive JPY 10,383.
Combination 8:	You will receive JPY 10,000 in 2 days.	After 9 days, you will receive JPY 10,574.
You were given a certain amount of money. You can get it after 90 or 97 days, but the amount will be different. If you had option A or B for the date and amount you would receive, which one would you choose? For combinations from 1 to 9, choose whichever you like and mark it with a circle.		
Question 2	Option A	Option B
Combination 1:	After 90 days, you will receive JPY 10,000.	After 97 days, you will receive JPY 9981.
Combination 2:	After 90 days, you will receive JPY 10,000.	After 97 days, you will receive JPY 10,000.
Combination 3:	After 90 days, you will receive JPY 10,000.	After 97 days, you will receive JPY 10,019.
Combination 4:	After 90 days, you will receive JPY 10,000.	After 97 days, you will receive JPY 10,038.
Combination 5:	After 90 days, you will receive JPY 10,000.	After 97 days, you will receive JPY 10,096.
Combination 6:	After 90 days, you will receive JPY 10,000.	After 97 days, you will receive JPY 10,191.
Combination 7:	After 90 days, you will receive JPY 10,000.	After 97 days, you will receive JPY 10,383.
Combination 8:	After 90 days, you will receive JPY 10,000.	After 97 days, you will receive JPY 10,574.

## Appendix B. Financial Knowledge, Behavior, and Attitude Questions

Financial Knowledge Questions						
1. Suppose you had JPY 100 in a savings account and the interest rate is 2% per year and you never withdraw money or interest payments. After 5 years, how much would you have in this account in total? (Check only one option)		1. More than JPY 102		2. Exactly JPY 102	3. Less than JPY 102	4. Do not know
2. Imagine that the interest rate on your savings account was 1% per year and inflation was 2% per year. After 1 year, how much would you be able to buy with the money in this account? (Check only one option)		1. More than today		2. Exactly the same	3. Less than today	4. Do not know
3. Please indicate whether the following statement is true or false (Choose only one): “Buying a company stock usually provides a safer return than a stock mutual fund”. (Check only one option)		1. True		2. False	3. Do not know	
Financial Behavior and Attitude Questions						
To what extent do the following statements apply to you? Please choose from 5 levels of degree. (Only one of each)	Applies		Somewhat True	Neither One nor the Other	Not Very Applicable	Not At All
Financial Behavior						
1. I carefully think before buying something.	1		2	3	4	5
2. I have never fallen behind in my payments.	1		2	3	4	5
3. I set long-term financial goals and strive to achieve them (financial behavior attitude).	1		2	3	4	5
4. I carefully spend/operate my money.	1		2	3	4	5
To what extent do the following statements apply to you? Please choose from 5 levels of degree. (Only one of each)	Applies		Somewhat True	Neither One nor the Other	Not Very Applicable	Not At All
Financial Attitude	1		2	3	4	5
1. I think it is more satisfying to spend money now than to save it for the future.	1		2	3	4	5
2. I tend to live for today and not think about tomorrow.	1		2	3	4	5

## Appendix C

**Table A1:** Correlation matrix.

Variables	(1)	(2)	(3)	(4)
(1) Financial Knowledge	1.000			
(2) Financial Behavior	0.190 *	1.000		
	(0.000)			
(3) Financial Attitude	0.152 *	0.360 *	1.000	
	(0.000)	(0.000)		
(4) Hyperbolic Discounting	−0.011 *	−0.035 *	−0.041 *	1.000
	(0.000)	(0.000)	(0.000)	

* *p* < 0.1.

**Table A2:** Variance inflation factor.

	Model 1	Model 2	Model 3	Model 4
Married	1.811	1.811	1.813	1.815
Household Income	1.52	1.512	1.511	1.521
Age	1.488	1.482	1.484	1.492
Household Assets	1.436	1.45	1.44	1.46
Children	1.396	1.396	1.396	1.397
Divorced	1.301	1.301	1.301	1.302
Employment	1.193	1.193	1.193	1.193
Financial Knowledge	1.121	-	-	1.158
University Education	1.12	1.105	1.102	1.121
Gender	1.107	1.068	1.069	1.112
Financial Behavior	-	1.031	-	1.19
Risk Aversion	1.029	1.03	1.029	1.03
Financial Attitude	-	-	1.029	1.179
Mean VIF	1.32	1.307	1.306	1.305
